# A Case with Spondyloenchondrodysplasia Treated with Growth Hormone

**DOI:** 10.3389/fendo.2017.00157

**Published:** 2017-07-10

**Authors:** Takanori Utsumi, Satoshi Okada, Kazushi Izawa, Yoshitaka Honda, Gen Nishimura, Ryuta Nishikomori, Rika Okano, Masao Kobayashi

**Affiliations:** ^1^Department of Pediatrics, Onomichi General Hospital, Hiroshima, Japan; ^2^Department of Pediatrics, Hiroshima University Graduate School of Biomedical & Health Sciences, Hiroshima, Japan; ^3^Department of Pediatrics, Kyoto University Graduate School of Medicine, Kyoto, Japan; ^4^Department of Pediatric Imaging, Tokyo Metropolitan Children’s Medical Center, Tokyo, Japan

**Keywords:** spondyloenchondrodysplasia, ACP5, growth hormone deficiency, growth hormone therapy, skeletal dysplasia

## Abstract

Spondyloenchondrodysplasia (SPENCD) is an autosomal recessive skeletal dysplasia caused by loss of function mutations in acid phosphatase 5, tartrate resistant (ACP5). Hypomorphic *ACP5* mutations impair endochondral bone growth and create an interferon (INF) signature, which lead to distinctive spondylar and metaphyseal dysplasias, and extraskeletal morbidity, such as neurological involvement and immune dysregulation, respectively. We report an affected boy with novel *ACP5* mutations, a splice-site mutation (736-2 A>C) and a nonsense mutation (R176X). He presented with postnatal short stature, which led to a diagnosis of partial growth hormone (GH) deficiency at 3 years of age. GH therapy was beneficial in accelerating his growth velocity. At 6 years of age, however, metaphyseal abnormalities of the knee attracted medical attention, and subsequent assessment ascertained the typical skeletal phenotype of SPENCD, brain calcifications, and an INF signature. This anecdotal experience indicates the potential efficacy of GH for growth failure in SPENCD.

## Introduction

Spondyloenchondrodysplasia (SPENCD, OMIM #607944) is a rare autosomal recessive skeletal dysplasia characterized by vertebral dysplasia and enchondroma-like radiolucent metaphyseal lesions of the long bones (1). Affected individuals manifest with short-trunked short stature. In addition, they show a variety of extraskeletal abnormalities, including neurological symptoms (intracranial calcification, developmental delay, spasticity, clumsy movements), and immune dysregulation (autoimmune diseases, immunodeficiency) (2–10). Recently, two groups reported biallelic hypomorphic mutations in the Acid Phosphatase 5, Tartrate Resistant (*ACP5*) gene as a genetic cause of SPENCD (11, 12). *ACP5* encodes tartrate-resistant acid phosphatase (TRAP). TRAP deficiency increases levels of phosphorylated osteopontin, which causes dysregulated endochondral ossification and other manifestations, and also an interferon (INF) signature (increased expression of type I INF regulated genes) leading to extraskeletal complications (11–13). Very little is documented concerning medical intervention for short stature and INF signature in SPENCD. Some SPENCD reports have mentioned attempts of growth hormone (GH) therapy, but the effect of GH has not been fully detailed (4, 10).

We report here an affected Japanese boy with novel compound heterozygous *ACP5* mutations, who had the typical skeletal phenotype of SPENCD, brain calcifications, absence of TRAP activity, and laboratory findings of an INF signature, but no immunodeficiency or autoimmune disorders. He had developed short stature in early childhood, leading to a diagnosis of partial GH deficiency (GHD) and introduction of GH therapy prior to the definitive diagnosis of SPENCD. Interestingly, his growth velocity was significantly accelerated in response to GH treatment.

## Case Report

**Figure 1:**
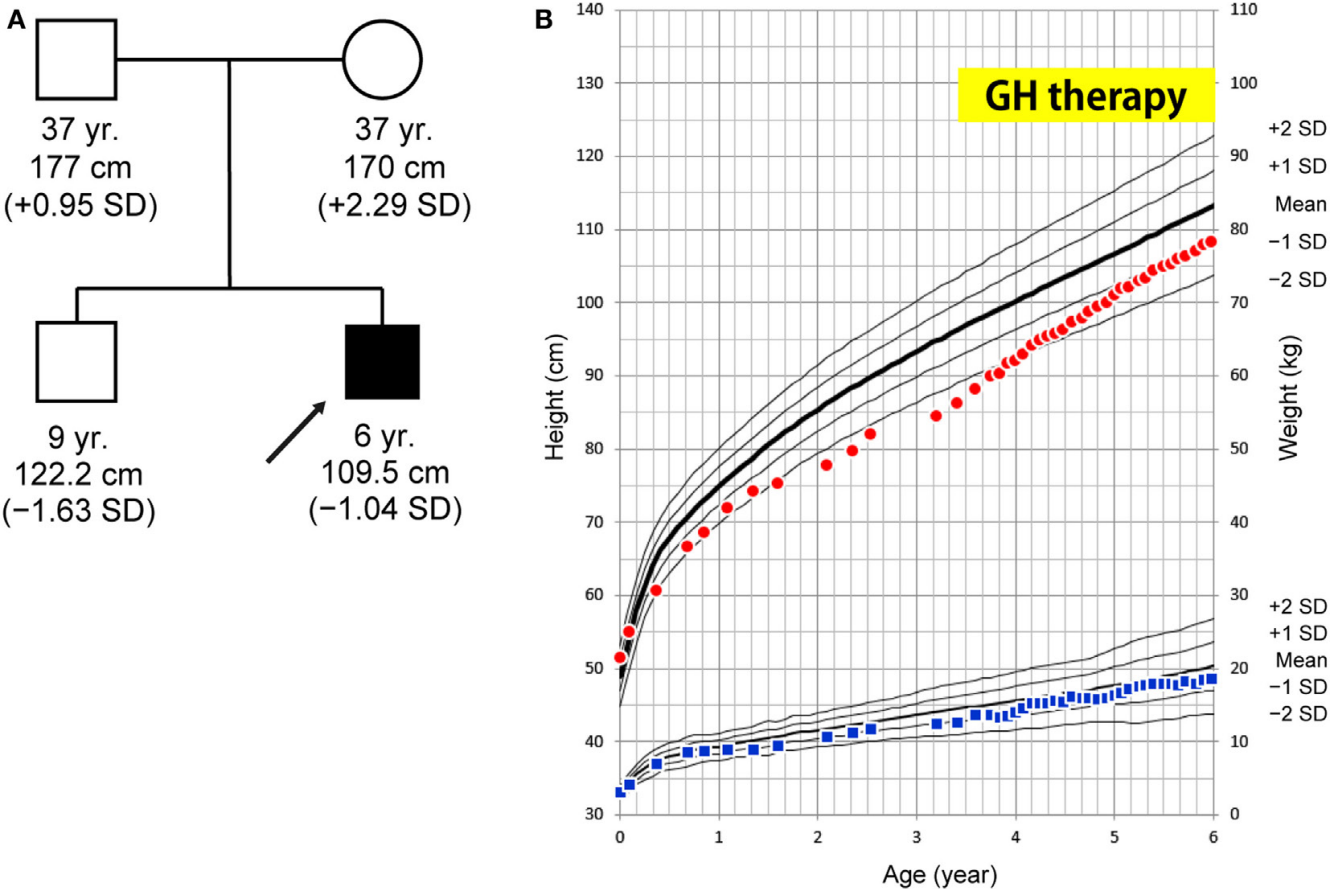
**(A)** Family tree. **(B)** Growth curve. At age 38 months, the patient’s height was 84.5 cm (−2.89 SD) and growth hormone (GH) therapy was commenced. The patient’s growth curve significantly improved with GH treatment over time. Height curve; red circles, weight curve; blue squares.

The patient is a 6-year-old Japanese boy who was born to non-consanguineous healthy parents. The patient’s mid-parental target height was 180 cm (final height of his father and mother was 177 and 170 cm, respectively). His older brother was healthy (Figure 1A). He was born at term after an uncomplicated pregnancy and delivery. Birth weight was 3,055 g (−0.64 SD), length 51.5 cm (+1.06 SD), and head circumference 34.0 cm (+0.40 SD). At 19 months, he was referred to us because of his inability to walk unaided. At that time brain MRI yielded a normal finding. He entered a rehabilitation program that improved his ambulation. His linear growth started to slow down from 5 months of age. At 38 months of age his height was 84.5 cm (−2.89 SD) (Figure 1B). Thyroid function was normal (TSH 4.520 μU/mL, FT4 1.06 ng/dL). The level of baseline insulin-like growth factor I (IGF-I) was low (18 ng/mL). These clinical manifestations and results of a GH stimulation test led to the diagnosis of partial GHD (peak GH response to arginine was 4.60 ng/mL and to clonidine was 2.41 ng/mL; the cut-off point was <6 ng/mL to define partial GHD and <3 ng/mL to define severe GHD) (Table S1 in Supplementary Material). GH therapy (0.175 mg/kg/week) was introduced, and it significantly accelerated his growth velocity (Figure 1B).

**Figure 2:**
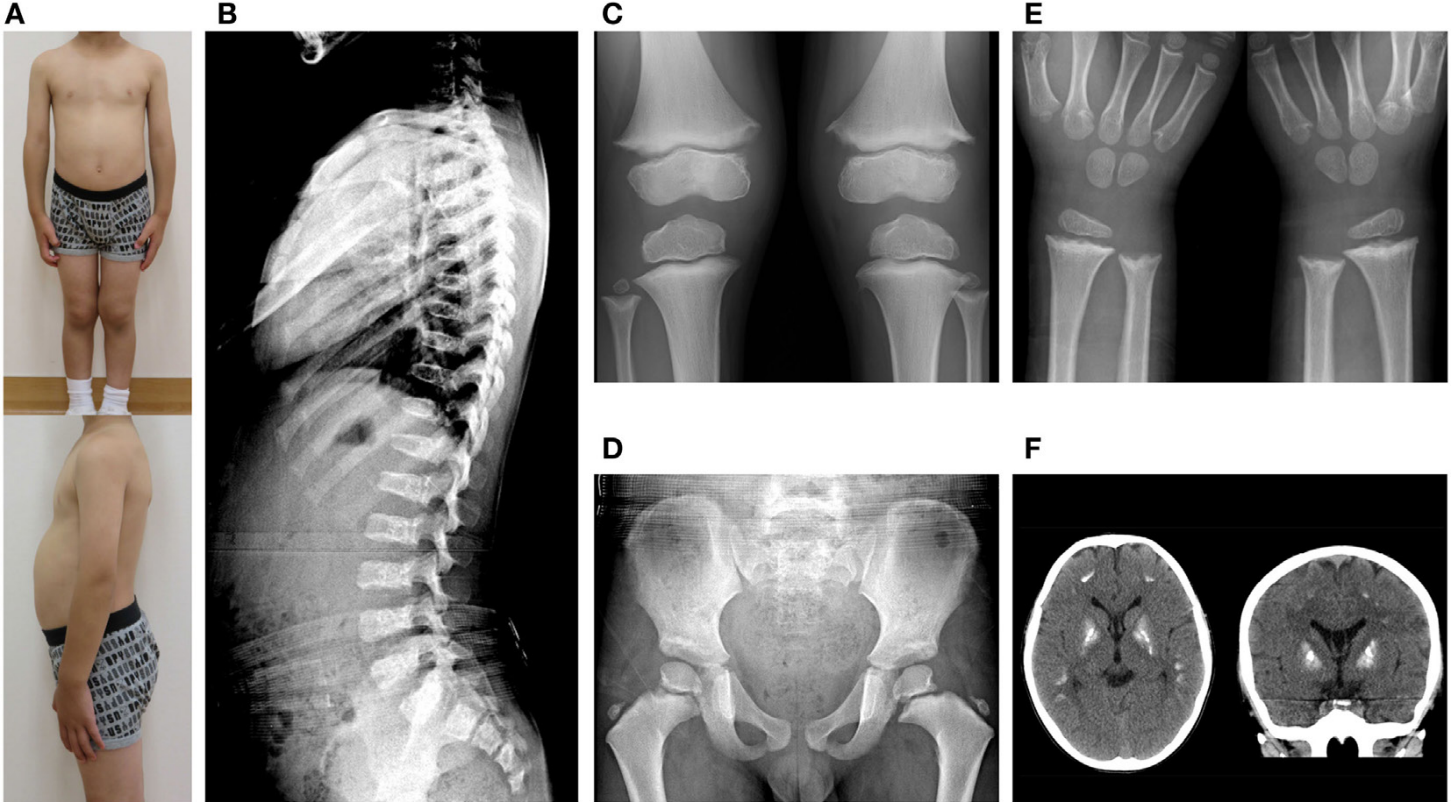
**(A)** Clinical photographs. **(B)** Spinal radiograph showing generalized platyspondyly with irregular endplates and irregular ossifications in the posterior vertebral bodies. **(C)** Radiograph of the knees showing irregular and sclerotic splayed metaphyses of the distal femur, proximal tibia, and fibula. **(D,E)** Radiograph of the hip and wrist showing similar metaphyseal changes as the knee. **(F)** Brain CT showing bilateral calcification of the basal ganglia.

At 6 years of age, he presented with painful knee joints. Height was 109.5 cm (−1.04 SD), and arm span was 111 cm. Facial features and body proportion were normal (Figure 2A). Development (language and cognitive skills, and fine and gross motor skills) was appropriate for his age, other than some clumsiness in motion. A radiograph of the knee showed mild metaphyseal dysplasia of the knee (shaved contour and marginal spur in the distal femoral metaphyses and to a lesser extent in the proximal tibial metaphyses, and mild cupping proximal fibular metaphyses) (Figure 2C). A subsequent skeletal survey revealed generalized metaphyseal dysplasia involving the hip and wrist as well as the knee (Figures 2D,E). The spine showed generalized platyspondyly with irregular endplates. The posterior vertebral bodies were irregularly ossified (Figure 2B). These radiographic findings raised a suspicion of SPENCD. Laboratory data showed normal levels of serum calcium, phosphate, alkaline phosphatase, intact PTH, IGF-I, and thyroid function (Table S1 in Supplementary Material). No immunological abnormality was found, including normal levels of complements and no detectable autoantibodies. TRAP activity was undetectable (TRAP-5b < 0.1 U/L). Bone mineral density on dual-energy x-ray absorptiometry was normal (L1–L4: 0.942 g/cm^2^). Brain CT of the patient showed bilateral calcifications of the basal ganglia (Figure 2F).

## Materials and Methods

### Genetic Analysis

The genetic study of the patient was performed using a candidate gene approach with Sanger sequencing of the *ACP5* gene. A familial study was not performed because consent was not given.

### INF Analysis

The expression of INF-stimulated genes in the patient’s whole blood cells were measured by qPCR as described previously (14). Whole blood was collected into PAXgene tubes (PreAnalytix, Hombrechtikon, Swizerland) and frozen at −20°C until extraction using the manufacturer’s protocol. Total RNA was extracted with a PAXgene RNA Blood kit (PreAnalytix) and reverse-transcribed to cDNA using an Omniscript RT kit (Qiagen, Hilden, Germany). Real time quantitative PCR was performed in triplicate using a 7900HT Fast Real-Time PCR system (Applied Biosystems, Waltham, MA, USA) and a kit for each INF-stimulated gene (*IFI27*, Hs01086370_m1; *IFIT1*, Hs00356631_g1; *RSAD2*, Hs01057264_m1; *SIGLEC1*, Hs00988063_m1; *ISG15*, Hs00192713_m1; *IFI44L*, Hs00199115_m1; *BACT*, Hs01060665_g1) (Life Technologies, Carlsbad, CA, USA). The expression levels were normalized to those of β-actin. Results are shown relative to a single calibrator (control 1).

## Results

### Identification of *ACP5* Mutations

**Figure 3:**
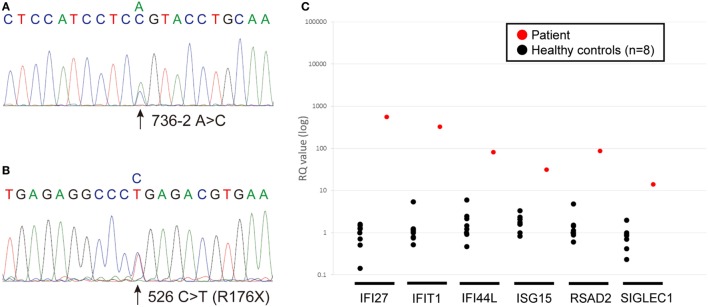
**(A,B)** Chromatograms showing the novel heterozygous mutations in *ACP5* identified in the patient; a splice-site mutation (c.736-2 A>C) **(A)** and a nonsense mutation (c.526 C>T; p.R176X) **(B)**. **(C)** Quantitative RT-PCR of INF-stimulated gene expression for, *IFI27, IFIT1, IFI44L, ISG15, RSAD2, SIGLEC1* in peripheral blood cells. The relative abundance of each transcript was normalized to the expression of β-actin. The experiment was performed in triplicate. Healthy control (*n* = 8); black circles, patient; red circles.

The sequence analysis revealed novel *ACP5* mutations, a splice-site mutation (c.736-2 A>C) and a nonsense mutation (c.526 C>T; p.R176X) (Figures 3A,B). Neither 736-2 A>C nor R176X were found in the NCBI, Ensembl, dbSNP, or ExAc databases.

### Detection of INF Signature

We identified marked upregulation of *IFI27, IFIT1, IFI44L, ISG15, RSAD2*, and *SIGLEC1* in the patient compared with healthy controls (*n* = 8) (Figure 3C).

## Discussion

We report a boy with a typical SPENCD skeletal phenotype, who presented with postnatal short stature and brain calcifications. He has not developed immune dysregulation despite laboratory evidence of a type I INF signature. An IFN signature in SPENCD plays a major role in neurological and immune involvement. We summarized the clinical manifestations of 48 patients with SPENCD by reviewing previous reports, as presented in Table 1 (1–9, 15–22). Height ranged from −1.5 to −7.5 SD (only cases with SD notation were included). The common features that first prompt the seeking of medical attention include musculoskeletal symptoms (60.4%), such as short stature, short limbs, kyphoscoliosis, and bowlegs, followed by neurological symptoms (27.1%) and immune dysregulation (18.8%). On the other hand, Briggs et al. (10) reviewed 26 patients, including 12 previously reported patients, and identified that up to 50% initially presented with immune dysregulation, followed by musculoskeletal symptoms (46.2%) and neurological symptoms (23.1%). This difference between our review and Briggs’ may represent the clinical diversity of SPENCD.

**Table 1:** Literature review of SPENCD case reports.

Author	Patient no.	Gender	At initial presentation		Growth failure	Neurological symptoms	Intracranial calcification	Immune dysregulation	Reference
			Age	Clinical symptoms					
Schorr et al.	Pt. 1	M	8 years	Short stature	Yes	No	ND	No	(1)
	Pt. 2	M	5 years	Short stature	Yes	No	ND	Recurrent infections	

Gustavson et al.	Pt. 3	F	15 months	Short stature	−7.5 SD at 16 years^a^	No	ND	ND	(15)
	Pt. 4	M	Birth	Short stature, short limbs, bowlegs	−6.5 SD at 13 years^a^	No	ND	ND	

Sauvegrain et al.	Pt. 5	F	16 years	Short stature, short neck	Yes	No	ND	ND	(16)
	Pt. 6	M	7 years	Short stature, Dev. delay	Yes	Dev. delay	ND	ND	

Chagnon et al.	Pt. 7	M	8 years	Spastic quadriplegia	Yes	Spasticity	ND	ND	(17)

Menger et al.	Pt. 8	M	Birth	Short limbs	−6 SD at 12 years	Dev. delay	ND	ND	(18)
	Pt. 9	M	Birth	Short limbs	−6 SD at 9 years	Dev. delay	ND	ND	
	Pt. 10	M	4 months	Craniotabes, costochondral beading	−1.5 SD at 12 years	No	ND	ND	
	Pt. 11	M	6 years	Short stature, spastic quadriplegia, kyphoscoliosis	−2 SD at 6 years	Dev. delay and spasticity	ND	ND	

Frydman et al.	Pt. 12	M	2.5 years	Short stature	−2.5 SD at 11 years	No	No	No	(2)
	Pt. 13	F	2 years	Short stature	−3.5 SD at 8 years	No	No	ND	
	Pt. 14	M	10 years	ND	ND	ND	ND	ND	
	Pt. 15	M	15 years	Short stature, kyphosis, pectus carinatum, spastic quadriparesis	−3 SD at 15 years	Spasticity	Yes	ND	
	Pt. 16	M	2 years	Walking difficulties, spastic quadriparesis	−4.4 SD at 8 years	Dev. delay and spasticity	Yes	ND	
	Pt. 17	M	18 years	Lack of ejaculate	−5.4 SD at 18 years	No	Yes	ND	

Robinson et al.	Pt. 18	M	11 years	Short stature, joint pain	<3rd percentile at 19 years	No	ND	ND	(19)
	Pt. 19	M	ND	ND	−5.5 SD at 86 years^a^	ND	ND	ND	

Uhlmann et al.	Pt. 20	M	Birth	Short stature, clubfoot, diastasis recti, low-set ears, simian creases, short neck	<3rd percentile at 5 years	No	ND	No	(20)
	Pt. 21	M	11 months	Short stature	Yes	No	ND	No	

Roifman and Melamed	Pt. 22	F	5 months	Recurrent infections	<3rd percentile at 18 years	No	ND	Recurrent infections, ITP, hypothyroidism, vitiligo	(3)
	Pt. 23	M	3 months	Recurrent infections	ND	No	No	Recurrent infections, ITP, hypothyroidism	
	Pt. 24	M	3 years	Recurrent infections	Yes	No	ND	Recurrent infections, ITP	
	Pt. 25	M	Infancy	Recurrent infections	Around 10th percentile at 10 years	No	ND	Recurrent infections, JRA, Crohn’s disease, hypothyroidism, vitiligo	

Tuysuz et al.	Pt. 26	M	10 years	Short stature	<3rd percentile at 10 years	No	No	ND	(4)
	Pt. 27	F	9 years	Short stature	<3rd–10th percentile at 21 years	No	No	ND	
	Pt. 28	M	7 years	Short stature, Dev. delay	<3rd percentile at 7 years	Dev. delay	Yes	ND	

Bhargava et al.	Pt. 29	M	7 years	Back pain, kyphosis	<5th percentile at 13 years	No	ND	No	(21)
	Pt. 30	F	Birth	Short stature	<5th percentile at 42 years	ND	ND	ND	

Renella et al.	Pt. 31	M	3 years	Short stature	<3rd percentile at 3 years	No	No	SLE	(5)
	Pt. 32	F	8 months	Leg stiffness	<3rd percentile at 3 years	Dev. delay and spasticity	Yes	No	
	Pt. 33	F	1 months	Short stature	Yes	No	ND	No	
	Pt. 34	M	1 months	Leg stiffness	<3rd percentile at 18 years	Dev. delay and spasticity	Yes	AIHA, FUO, ITP	
	Pt. 35	M	21 months	Leg stiffness, spastic paraparesis	Yes	Dev. delay and spasticity	ND	ITP, AIHA	
	Pt. 36	M	10 years	Recurrent thrombocytopenia, recurrent fever of unknown origin	<3rd percentile at 17 years	Dev. delay and spasticity	Yes	ITP, FUO	
	Pt. 37	M	Childhood	Short stature	Yes	Spasticity	ND	ND	
	Pt. 38	F	11 years	Rheumatic fever with carditis, sydenham chorea	<3rd percentile at 12 years	No	ND	Rheumatic fever	
	Pt. 39	M	8 months	Short stature	<3rd percentile at 7 years	Dev. delay and spasticity	No	Inflammatory syndrome with hypogammaglobulinemia, AIHA	

Kulkarni et al.	Pt. 40	M	2 years	Recurrent infections	<5th percentile at 5 years	No	ND	Recurrent infections, compromised cellular immunity, SLE, ITP, JRA	(6)

Navarro et al.	Pt. 41	F	3 years	Febrile seizures	<5th percentile at 23 years	Dev. delay	Yes	Sjögren syndrome, polymyositis, hypothyroidism, pancreatitis, autoimmune multifocal neuropathy, scleroderma	(7)
	Pt. 42	M	2.5 years	Walking difficulties, skin rash	<5th percentile at 10 years	Spasticity	Yes	Leukocytoclastic vasculitis, SLE	

Girschick et al.	Pt. 43	F	6 months	Increased muscle tone of the lower limbs	Yes	Spasticity	No	AIHA, ITP, polyarthritis, hepatitis, nephritis, life-threatening hyperreactivity to viral infections as well as recurrent bacterial infections	(8)

de Bruin et al.	Pt. 44	F	13 years	Short stature	−5.5 SD at 13 years	Dev. delay	No	No	(22)
	Pt. 45	M	8 years	Short stature	−5.1 SD at 8 years	Dev. delay	No	No	

Bilginer et al.	Pt. 46	M	5 years	Short stature	<3rd percentile at 5 years	ND	Yes	SLE (lupus nephritis)	(9)
	Pt. 47	F	16 years	Short stature, arthralgia, arthritis	<3rd percentile at 16 years	ND	Yes	SLE (lupus nephritis), vitiligo	
	Pt. 48	F	4.5 years	Leg stiffness, walking difficulties	<3rd percentile at 16 years	Spasticity	Yes	SLE (lupus nephritis)	

*M, male; F, female; ND, not described; Dev. delay, developmental delay; ITP, idiopathic thrombocytopenic purpura; JRA, juvenile rheumatoid arthritis; SLE, systemic lupus erythematosus; AIHA, autoimmune hemolytic anemia; FUO, fever of unknown origin*.

*^a^Described in Ref. (10)*.

In the present case, postnatal growth failure was ameliorated with GH therapy; height increased from −2.89 SD to −1.04 SD over 3 years. The patient’s IGF-I levels increased to within the normal range after commencing GH therapy (Figure S1 in Supplementary Material). In addition, GH therapy did not accelerate bone age; bone ages were 2.3 and 5.2 years at the chronological ages of 3 years 4 months and 6 years 11 months, respectively. These findings indicate the potential therapeutic benefit of GH to improve short stature in patients with SPENCD.

Two reports have been published describing three SPENCD patients who underwent GH therapy (4, 10). Briggs et al. (10) described two patients, including one patient with GHD who responded well to GH therapy. Tuysuz et al. (4) reported a 21-year-old woman with SPENCD who received GH therapy for 2 years from the age of 9 years such that she achieved a normal height (153 cm: 3–10 centile). It remains unclear whether a good response to GH therapy in these SPENCD patients is universal or not. Lack of TRAP leads to an increase in phosphorylated osteopontin, which is responsible for disordered endochondral ossification and reduced resorption of calcified cartilage matrix and primary spongiosa as a result of impaired adhesion, migration, and activation of osteoclasts (11, 13, 23, 24). Meanwhile, GH advances longitudinal bone growth directly by stimulating prechondrocyte differentiation, and indirectly by clonal expansion of differentiated chondrocytes through upregulation of IGF-I (25–29). Moreover, GH stimulates resorption of cartilage matrix and immature bone through both its direct and indirect actions on osteoclast formation and differentiation and through indirect activation of mature osteoclasts *via* osteoblasts. Upregulation of IGF-I associated with GH therapy also supports osteoclastic activities (30, 31). These effects of GH may efficiently counteract the negative effects on endochondral bone growth in SPENCD.

Growth hormone therapy is associated with adverse risks in some patients, such as femoral head necrosis, slipped capital femoral epiphysis, exostosis, and the progression of bone deformities, particularly spinal deformities, such as scoliosis. We have not observed any obvious adverse events associated with GH therapy in the present case up to this time. However, because only 3 years has passed since the patient commenced GH therapy, the patient will require regular follow-up to evaluate the potential long-term effects of GH therapy. In addition, there are some other limitations in our study. First, the patient has partial GHD as well as SPENCD, and therefore the potential benefit of GH therapy for short stature in patients with SPENCD might be overestimated. Second, the patient is 6 years old and is too young to evaluate the effect of GH therapy on his final height. Despite these limitations, however, the improvement in growth velocity found in the present case is encouraging and may suggest the potential therapeutic benefit of GH in patients with SPENCD.

## Concluding Remarks

This case report described the treatment of a patient who presented progressive growth failure associated with SPENCD and partial GHD. After commencing GH therapy, the patient’s height SD score improved from −2.89 SD to −1.04 SD over 3 years without accelerating the bone age. Considering the improvement in growth velocity in the present case, it is reasonable to suggest that GH therapy is warranted in patients with SPENCD, at least for those who show evidence of a partial GHD. Further studies are recommended to evaluate the benefit of GH therapy in improving the height of patients with SPENCD, even in the absence of GHD.

## Ethics Statement

This study was approved by the Institutional Review Board of Kyoto University. We obtained written informed consent for genomic and interferon analysis of the patient from the parents in accordance with the Declaration of Helsinki. Also, the parents of the patient provided written informed consent for the publication of the patient’s identifiable information.

## Author Contributions

Patient workup: TU. Interpretation of radiographs: GN. Genetic and IFN signature analysis: KI, YH, and RN. Interpretation of data, drafting the manuscript or critical revision, final approval of the version to be published, and agreement to be accountable for all aspects of the work: TU, SO, KI, YH, GN, RN, RO, and MK.

## Conflict of Interest Statement

The authors declare that the research was conducted in the absence of any commercial or financial relationships that could be construed as a potential conflict of interest.

## References

[1] Schorr S Legum C Ochshorn M. Spondyloenchondrodysplasia. Enchondromatomosis with severe platyspondyly in two brothers. Radiology (1976) 118(1):133–9. 10.1148/118.1.133 1244645

[2] Frydman M Bar-Ziv J Preminger-Shapiro R Brezner A Brand N Ben-Ami T Possible heterogeneity in spondyloenchondrodysplasia: quadriparesis, basal ganglia calcifications, and chondrocyte inclusions. Am J Med Genet (1990) 36(3):279–84. 10.1002/ajmg.1320360306 2363422

[3] Roifman CM Melamed I. A novel syndrome of combined immunodeficiency, autoimmunity and spondylometaphyseal dysplasia. Clin Genet (2003) 63(6):522–9. 10.1034/j.1399-0004.2003.00033.x 12786759

[4] Tuysuz B Arapoglu M Ungur S. Spondyloenchondrodysplasia: clinical variability in three cases. Am J Med Genet A (2004) 128A(2):185–9. 10.1002/ajmg.a.30078 15214014

[5] Renella R Schaefer E LeMerrer M Alanay Y Kandemir N Eich G Spondyloenchondrodysplasia with spasticity, cerebral calcifications, and immune dysregulation: clinical and radiographic delineation of a pleiotropic disorder. Am J Med Genet A (2006) 140(6):541–50. 10.1002/ajmg.a.31081 16470600

[6] Kulkarni ML Baskar K Kulkarni PM A syndrome of immunodeficiency, autoimmunity, and spondylometaphyseal dysplasia. Am J Med Genet A (2007) 143A(1):69–75. 10.1002/ajmg.a.31526 17163538

[7] Navarro V Scott C Briggs TA Barete S Frances C Lebon P Two further cases of spondyloenchondrodysplasia (SPENCD) with immune dysregulation. Am J Med Genet A (2008) 146A(21):2810–5. 10.1002/ajmg.a.32518 18924170

[8] Girschick H Wolf C Morbach H Hertzberg C Lee-Kirsch MA. Severe immune dysregulation with neurological impairment and minor bone changes in a child with spondyloenchondrodysplasia due to two novel mutations in the ACP5 gene. Pediatr Rheumatol Online J (2015) 13(1):37. 10.1186/s12969-015-0035-7 26346816 PMC4562156

[9] Bilginer Y Duzova A Topaloglu R Batu ED Boduroglu K Gucer S Three cases of spondyloenchondrodysplasia (SPENCD) with systemic lupus erythematosus: a case series and review of the literature. Lupus (2016) 25(7):760–5. 10.1177/0961203316629000 26854080

[10] Briggs TA Rice GI Adib N Ades L Barete S Baskar K Spondyloenchondrodysplasia due to mutations in ACP5: a comprehensive survey. J Clin Immunol (2016) 36(3):220–34. 10.1007/s10875-016-0252-y 26951490 PMC4792361

[11] Lausch E Janecke A Bros M Trojandt S Alanay Y De Laet C Genetic deficiency of tartrate-resistant acid phosphatase associated with skeletal dysplasia, cerebral calcifications and autoimmunity. Nat Genet (2011) 43(2):132–7. 10.1038/ng.749 21217752

[12] Briggs TA Rice GI Daly S Urquhart J Gornall H Bader-Meunier B Tartrate-resistant acid phosphatase deficiency causes a bone dysplasia with autoimmunity and a type I interferon expression signature. Nat Genet (2011) 43(2):127–31. 10.1038/ng.748 21217755 PMC3030921

[13] Behrens TW Graham RR TRAPing a new gene for autoimmunity. Nat Genet (2011) 43(2):90–1. 10.1038/ng0211-90 21270835

[14] Oda H Nakagawa K Abe J Awaya T Funabiki M Hijikata A Aicardi-Goutieres syndrome is caused by IFIH1 mutations. Am J Hum Genet (2014) 95(1):121–5. 10.1016/j.ajhg.2014.06.007 24995871 PMC4085581

[15] Gustavson KH Holmgren G Probst F. Spondylometaphyseal dysplasia in two sibs of normal parents. Pediatr Radiol (1978) 7(2):90–6. 10.1007/BF00975677 673535

[16] Sauvegrain J Maroteaux P Ribier J Garel L Tato L Rochiccioli P [Multiple chondroma affecting the spine: spondylo-enchondroplasia and other forms (author’s transl)]. J Radiol (1980) 61(8–9):495–501. 7463391

[17] Chagnon S Lacert P Blery M. [Spondylo-enchondrodysplasia]. J Radiol (1985) 66(1):75–7. 3999058

[18] Menger H Kruse K Spranger J. Spondyloenchondrodysplasia. J Med Genet (1989) 26(2):93–9. 10.1136/jmg.26.2.93 2918547 PMC1015557

[19] Robinson D Tieder M Copeliovitch L Halperin N Spondyloenchondrodysplasia. A rare cause of short-trunk syndrome. Acta Orthop Scand (1991) 62(4):375–8. 1882681 10.3109/17453679108994474

[20] Uhlmann D Rupprecht E Keller E Hormann D Spondyloenchondrodysplasia: several phenotypes – the same syndrome. Pediatr Radiol (1998) 28(8):617–21. 10.1007/s002470050431 9716637

[21] Bhargava R Leonard NJ Chan AK Spranger J. Autosomal dominant inheritance of spondyloenchondrodysplasia. Am J Med Genet A (2005) 135(3):282–8. 10.1002/ajmg.a.30732 15887273

[22] de Bruin C Orbak Z Andrew M Hwa V Dauber A. Severe short stature in two siblings as the presenting sign of ACP5 deficiency. Horm Res Paediatr (2016) 85(5):358–62. 10.1159/000443684 26789720 PMC4891295

[23] Hayman AR Jones SJ Boyde A Foster D Colledge WH Carlton MB Mice lacking tartrate-resistant acid phosphatase (Acp 5) have disrupted endochondral ossification and mild osteopetrosis. Development (1996) 122(10):3151–62. 8898228 10.1242/dev.122.10.3151

[24] Janckila AJ Yam LT. Biology and clinical significance of tartrate-resistant acid phosphatases: new perspectives on an old enzyme. Calcif Tissue Int (2009) 85(6):465–83. 10.1007/s00223-009-9309-8 19915788

[25] Isaksson OG Jansson JO Gause IA. Growth hormone stimulates longitudinal bone growth directly. Science (1982) 216(4551):1237–9. 10.1126/science.7079756 7079756

[26] Green H Morikawa M Nixon T. A dual effector theory of growth-hormone action. Differentiation (1985) 29(3):195–8. 10.1111/j.1432-0436.1985.tb00316.x 3908201

[27] Zezulak KM Green H The generation of insulin-like growth factor-1 – sensitive cells by growth hormone action. Science (1986) 233(4763):551–3. 10.1126/science.3726546 3726546

[28] Isaksson OG Lindahl A Nilsson A Isgaard J Mechanism of the stimulatory effect of growth hormone on longitudinal bone growth. Endocr Rev (1987) 8(4):426–38. 10.1210/edrv-8-4-426 3319530

[29] Isgaard J Moller C Isaksson OG Nilsson A Mathews LS Norstedt G. Regulation of insulin-like growth factor messenger ribonucleic acid in rat growth plate by growth hormone. Endocrinology (1988) 122(4):1515–20. 10.1210/endo-122-4-1515 3345724

[30] Nishiyama K Sugimoto T Kaji H Kanatani M Kobayashi T Chihara K. Stimulatory effect of growth hormone on bone resorption and osteoclast differentiation. Endocrinology (1996) 137(1):35–41. 10.1210/endo.137.1.8536635 8536635

[31] Mochizuki H Hakeda Y Wakatsuki N Usui N Akashi S Sato T Insulin-like growth factor-I supports formation and activation of osteoclasts. Endocrinology (1992) 131(3):1075–80. 10.1210/endo.131.3.1505451 1505451

