# Supplementary material for: A Case with Spondyloenchondrodysplasia Treated with Growth Hormone
Source: Front Endocrinol (Lausanne). 2017 Jul 10;8:157. doi: 10.3389/fendo.2017.00157 (PMC5502255; doi:10.3389/fendo.2017.00157)
Supplement: Supplementary file 1 [file Data_Sheet_1.DOCX]

Supplementary Material

A Case with Spondyloenchondrodysplasia Treated with Growth Hormone

Takanori Utsumi^1*^, Satoshi Okada^2^, Kazushi Izawa^3^, Yoshitaka Honda^3^, Gen Nishimura^4^, Ryuta Nishikomori^3^, Rika Okano^1^, Masao Kobayashi^2^

*** Correspondence:**Takanori Utsumi
[t.utsumi.s15@gmail.com](mailto:t.utsumi.s15@gmail.com)

# Supplementary Table

# GH stimulation test (at the age of 3 years)

| **Arginine tolerance test** | **Basal** | **30 min** | **60 min** | **90 min** | **120 min** |
| --- | --- | --- | --- | --- | --- |
| GH (ng/mL) | 1.98 | 1.54 | 3.64 | 4.60 | 2.62 |
|  |  |  |  |  |  |
| **Clonidine tolerance test** | **Basal** | **30 min** | **60 min** | **90 min** | **120 min** |
| GH (ng/mL) | 2.27 | 1.61 | 1.98 | 2.41 | 1.70 |

Biochemical/Hormonal data (at the age of 6 years)

| **Parameters** | **Patient** | **Reference range** |
| --- | --- | --- |
| Ca (mg/dL) | 9.2 | 8.7–10.2 |
| IP (mg/dL) | 5.1 | 3.9–5.8 |
| ALP (U/L) | 629 | 440–1230 |
| intact-PTH (pg/mL) | 16 | 10–65 |
| IGF-I (ng/mL) | 120 | 55–215 |
| TSH (µU/mL) | 4.335 | 0.430–4.000 |
| FT4 (ng/dL) | 1.03 | 1.03–2.00 |

# Supplementary Figure


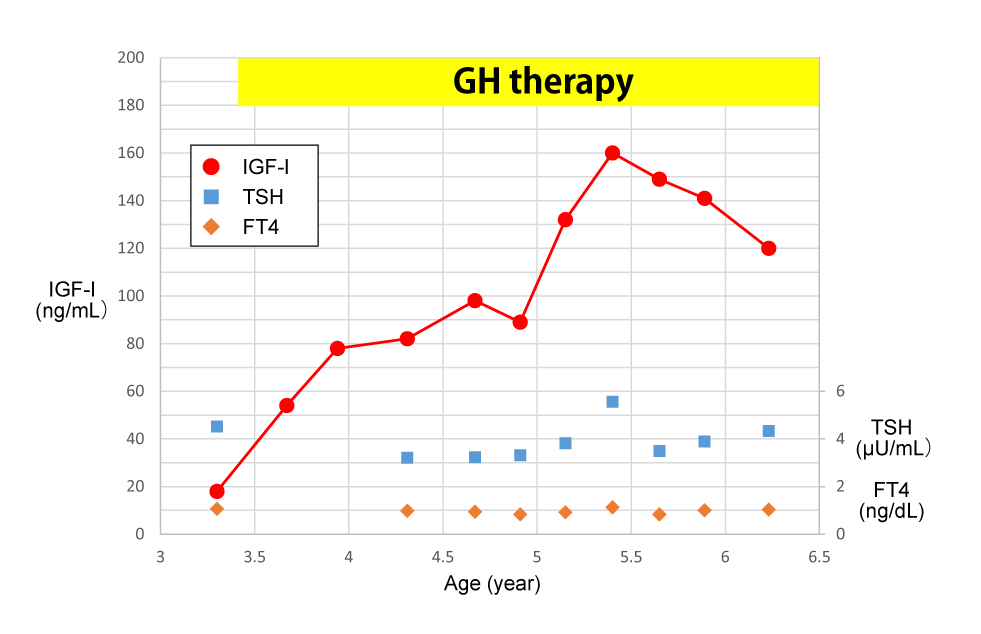
**Supplementary Figure.** Chronological changes in IGF-I, TSH, and FT4 levels. The IGF-I level increased to within normal levels after the commencement of GH therapy. The improvement in the patient’s growth (Figure 1B) coincided with the increase in IGF-I levels. TSH and FT4 levels were almost within the normal range.
